# Supplementary figures and images for: Comparative Genomics of Emerging Lineages and Mobile Resistomes of Contemporary Broiler Strains of Salmonella Infantis and E. coli
Source: Front Microbiol. 2021 Feb 25;12:642125. doi: 10.3389/fmicb.2021.642125 (PMC7947892; doi:10.3389/fmicb.2021.642125)

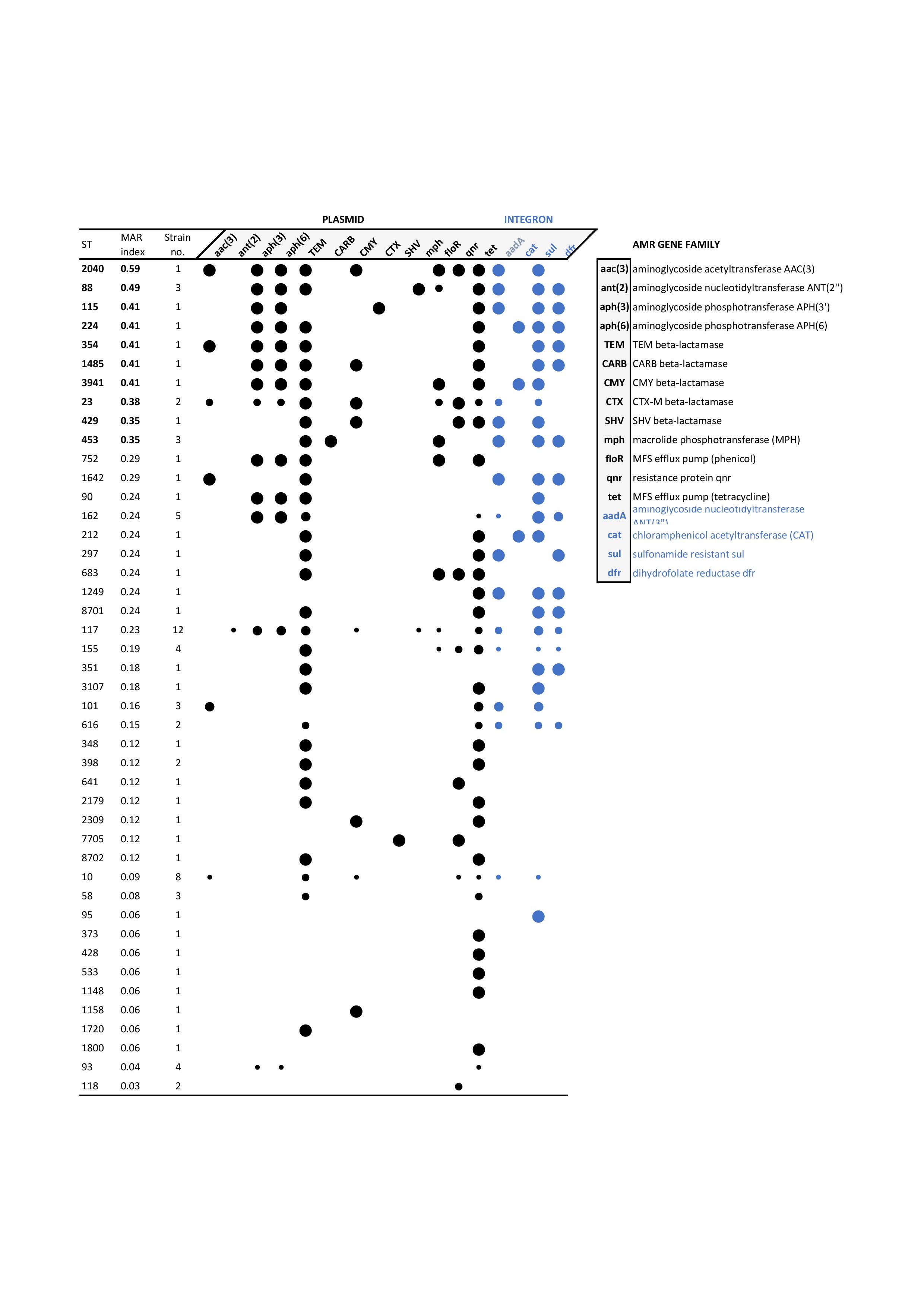

Supplement: Supplementary Figure 1 — Relation between the sequence type (ST) and abundance of acquired resistance genes. In bold are indicated STs that are potentially associated with increased abundance of acquired resistance genes (MAR index > 0.3). Circle sizes are proportional to the prevalence (%) of the given gene for each ST. “Empty” STs (ST46, 349, 355, and 665) are not presented. [file Image_1.JPEG]

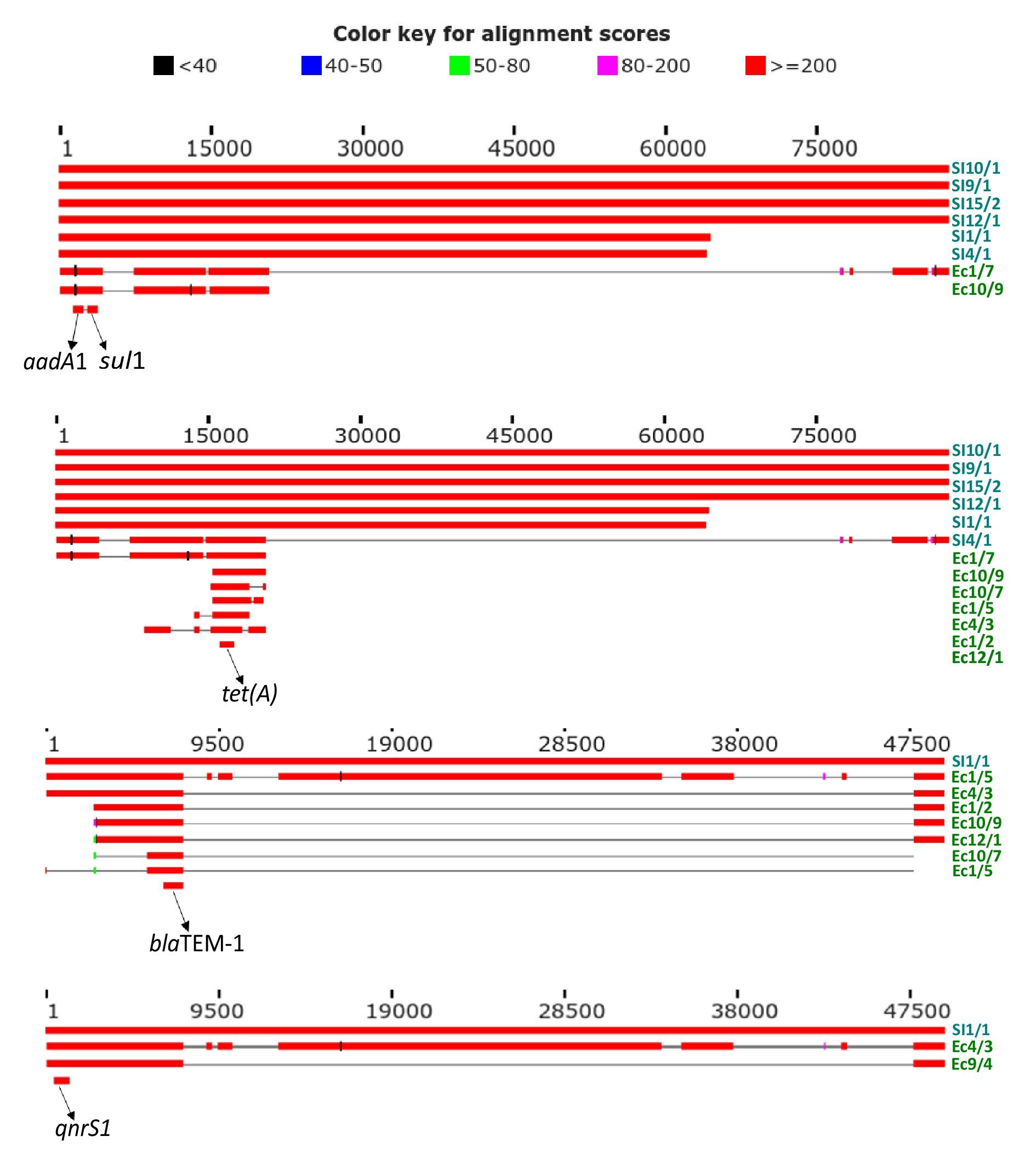

Supplement: Supplementary Figure 2 — Multiple sequence alignment of plasmid contigs carrying antibiotic resistance genes that overlap between cohabitant strains of S. Infantis and E. coli. The sequence identity of plasmid contigs including the aadA1, sul1, tet(A), blaTEM–1, or qnrS1 genes was compared between cohabitant S. Infantis and E. coli strains. S. Infantis strains are coloured in blue, while green represents E. coli strains. [file Image_2.JPEG]
